# Acknowledgement of manuscript reviewers 2015

**DOI:** 10.1186/s12954-016-0098-x

**Published:** 2016-02-26

**Authors:** Nick Crofts, Euan Lawson

**Affiliations:** The Melbourne School of Population and Global Health, Melbourne, Australia; Lancaster University, Lancaster, UK

## Abstract

The editors of *Harm Reduction Journal* would like to thank all our reviewers who have contributed to the journal in Volume 12 (2015).

**Robert Ali**

Australia

**Steve Allsop**

Australia

**Tasnim Azim**

Bangladesh

**Cas Barendregt**

Netherlands

**Brittany Barker**

Canada

**Andrew Barnes**

USA

**Angela Bazzi**

USA

**Lynne Belle-Isle**

Canada

**Ricky Bluthenthal**

USA

**Stephan Bongard**

Germany

**Susan Boyd**

Canada

**Jamie Brown**

UK

**Joanne Bryant**

Australia

**Brent Caldwell**

New Zealand

**Tessa Cheng**

Canada

**Stephen Corson**

UK

**Constanza Daigre**

Spain

**Lucia D'ambruoso**

UK

**Caitlin Davey**

Canada

**Peter Davidson**

USA

**Colleen Dell**

Canada

**John Derricott**

UK

**Don Des Jarlais**

US

**Elinor Dickie**

UK

**Ernest Drucker**

USA

**Nadia Fairbairn**

Canada

**Michael Farrell**

Australia

**Gabriele Fischer**

Austria

**Niamh Fitzgerald**

UK

**Chris Ford**

UK

**Vivian Go**

USA

**Shira Goldenberg**

USA

**Lauretta Grau**

USA

**Mauro Guarinieri**

Vietnam

**Andrew Guise**

UK

**Wayne Hall**

Australia

**Theodore Hammett**

USA

**Magdalena Harris**

UK

**Kanna Hayashi**

Canada

**Evelyn Hearne**

Ireland

**Peter Higgs**

Australia

**Martin Iguchi**

USA

**Ehsan Jozaghi**

Canada

**Bengt Kayser**

Switzerland

**Ken Kirkwood**

Canada

**Irma Kirtadze**

Georgia

**Andrea Krüsi**

Canada

**M Suresh Kumar**

India

**Pritika Kumar**

US

**Margot Kuo**

Canada

**Jih-Heng Li**

Taiwan

**Tim Mackey**

USA

**Olivier Maguet**

France

**Jagadish Mahanta**

India

**Marcus Martens**

Germany

**Catriona Matheson**

UK

**Andrew Mcauley**

UK

**Peter Meylakhs**

Russian Federation

**Ingo Michels**

Germany

**Alison Munro**

UK

**Bronwyn Myers**

South Africa

**David A L Newcombe**

New Zealand

**Robert Newman**

USA

**Suzanne Nielsen**

Australia

**Daniel O'Keefe**

Australia

**David Otiashvili**

Georgia

**Stephen Pan**

Canada

**Dana Paquette**

Canada

**Vibhu Paudyal**

UK

**Einat Peles**

Israel

**Christiane Pflanz-Sinclair**

UK

**Carl Phillips**

USA

**Eileen Pitpitan**

USA

**Riccardo Polosa**

Italy

**Seyed Radfar Ramin**

Iran

**Afarin Rahimi-Movaghar**

Iran

**Sanjeev Raj Neupane**

Nepal

**Teresita Rocha**

Mexico

**Elise Roy**

Canada

**Riza Sarasvita**

Indonesia

**Andrew Sceibe**

South Africa

**Sivasubramaniam Selvaraj**

UK

**Janie Sheridan**

New Zealand

**Andrew Smirnov**

Australia

**Richard Steen**

Italy

**Carol Strike**

Canada

**Victoria Sublette**

Australia

**Ave Talu**

Estonia

**Andy Tan**

USA

**Johannes Thrul**

USA

**Lianping Ti**

Canada

**Ambros Uchtenhagen**

Switzerland

**Lianne Urada**

US

**Edwin Van Teijlingen**

UK

**Jack Wallace**

Australia

**Nick Walsh**

Australia

**Brooke West**

USA

**Lucas Wiessing**

Portugal

**Alex Wodak**

Australia

**Heather Worth**

Australia

**Cheng-Fang Yen**

Taiwan

**Oleg Yussopov**

Kazakhstan

